# Alterations in Glucagon Levels and the Glucagon-to-Insulin Ratio in Response to High Dietary Fat or Protein Intake in Healthy Lean Adult Twins: A Post Hoc Analysis †

**DOI:** 10.3390/nu16223905

**Published:** 2024-11-15

**Authors:** Bettina Schuppelius, Rita Schüler, Olga Pivovarova-Ramich, Silke Hornemann, Andreas Busjahn, Jürgen Machann, Michael Kruse, Soyoung Q. Park, Stefan Kabisch, Marta Csanalosi, Anne-Cathrin Ost, Andreas F. H. Pfeiffer

**Affiliations:** 1 Department of Endocrinology and Metabolism, Charité—Universitätsmedizin Berlin, Corporate Member of Freie Universität Berlin and Humboldt-Universität zu Berlin, Charitéplatz 1, 10117 Berlin, Germany; 2 Department of Clinical Nutrition, German Institute of Human Nutrition Potsdam-Rehbruecke, Arthur-Scheunert-Allee 114-116, 14558 Nuthetal, Germany; 3 Department of Molecular Metabolism and Precision Nutrition, German Institute of Human Nutrition Potsdam-Rehbruecke, Arthur-Scheunert-Allee 114-116, 14558 Nuthetal, Germany; 4 German Center for Diabetes Research (DZD), Ingolstädter Landstrasse 1, 85764 München-Neuherberg, Germany; 5 HealthTwiSt GmbH, Robert-Rössle-Strasse 10, 13125 Berlin, Germany; 6 Institute of Diabetes Research and Metabolic Diseases (IDM) of the Helmholtz Centre Munich, Otfried-Müller-Str. 10, 72076 Tübingen, Germany; 7 Section of Experimental Radiology, Department of Diagnostic and Interventional Radiology, University of Tübingen, Geissweg 3, 72076 Tübingen, Germany; 8 Department of Decision Neuroscience and Nutrition, German Institute of Human Nutrition Potsdam-Rehbrücke, Arthur-Scheunert-Allee 114-116, 14558 Nuthetal, Germany; 9 Neuroscience Research Center, Charité—Universitätsmedizin Berlin, Corporate Member of Freie Universität Berlin, Humboldt-Universität zu Berlin, and Berlin Institute of Health, Charitéplatz 1, 10117 Berlin, Germany

**Keywords:** glucagon, insulin, high-fat diet, low-fat diet, high-protein diet, heritability, healthy twins

## Abstract

**Background/Objectives**: Emerging data support evidence of the essential role of glucagon for lipid metabolism. However, data on the role of dietary fat intake for glucagon secretion is limited. This analysis investigated whether altering nutritional fat intake affects glucagon levels in healthy subjects. **Methods**: A total of 92 twins (age: 31 ± 14 years, BMI: 23 ± 3 kg/m^2^) consumed two 6-week diets: first a low-fat, high-carbohydrate diet (LFD) followed by an isocaloric high-fat, low-carbohydrate diet (HFD). In total, 24 twins (age: 39 ± 15 years, BMI: 24 ± 2 kg/m^2^) continued with a high-protein diet (HPD). Clinical investigations were performed after 6 weeks of the LFD, after 1 and 6 weeks of the HFD and after 6 weeks of the HPD. **Results**: The LFD caused a significant decrease in fasting glucagon (−27%, *p* < 0.001) compared to baseline. After 6 weeks of the HFD, glucagon increased (117%, *p* < 0.001 vs. LFD), while free fatty acids decreased. Six weeks of the HPD further increased glucagon levels (72%, *p* = 0.502 vs. HFD), although fasting amino acid levels remained constant. Fasting insulin and HOMA-IR moderately increased after one week of the HFD, while six weeks of the HPD significantly decreased both. The fasting glucagon-to-insulin ratio decreased during the LFD (*p* < 0.001) but increased after the HFD (*p* < 0.001) and even further increased after the HPD (*p* = 0.018). Liver fat, triglycerides and blood glucose did not increase during the HFD. The heritability of glucagon levels was 45% with the LFD. **Conclusions**: An HFD increases glucagon levels and the glucagon-to-insulin ratio under isocaloric conditions compared to an LFD in healthy lean subjects. This rise in glucagon may represent a metabolic response to prevent hepatic steatosis, as glucagon increases have been previously shown to induce hepatic fat oxidation.

## 1. Introduction

Glucagon levels are closely related to insulin levels in healthy subjects, which together control fasting and postprandial plasma glucose levels in an intra-islet interplay of alpha and beta cells [1,2,3]. Glucagon increases hepatic glucose production by glycogenolysis or gluconeogenesis in the fasting state to prevent hypoglycemia [4]. Insulin was shown to suppress glucagon postprandially as reflected by an inverse relationship of insulin to glucagon levels in humans [1,2] and experimental systems [3,4]. Nevertheless, alpha-cell glucagon was recently shown to stimulate insulin release from beta cells through actions on beta-cell glucagon and GLP1 receptors which was required for intact beta-cell function in mice [4,5].

Glucose acutely suppresses glucagon levels in healthy subjects after oral intake or intravenous application [6], whereas amino acids are strong secretagogues for glucagon and were identified as powerful stimulators of alpha-cell proliferation [7,8]. In reverse, glucagon stimulates amino acid degradation by the urea cycle in the liver which plays a primary role in maintaining physiological amino acid levels [9,10,11]. Inhibition of glucagon signaling by antagonists or glucagon-receptor deletion leads to hyperaminoacidemia, which further induces the proliferation of alpha cells [7], while excess glucagon due to glucagonomas reduces circulating amino acid levels.

Emerging data indicate that glucagon is moreover involved in lipid metabolism since glucagon receptor antagonists were found to increase liver fat accumulation and LDL levels in early-phase clinical trials [12,13]. Glucagon was shown to increment hepatic lipolysis and hepatic ß-oxidation in mice, rats and humans via stimulation of the inositol trisphosphate receptor (INSP3R), thereby reversing hepatic steatosis in rodent models [14,15]. The question of whether and how lipids conversely regulate the secretion of glucagon is still debated. Free fatty acids (FFA) were shown to stimulate glucagon secretion in isolated alpha cells, which may function directly via the activation of FFA receptors, such as the G-protein-coupled receptor 40 (GPR40) and the GPR119, or indirectly via increased fatty acid oxidation in pancreatic islets [13,16,17]. Mice fed a high-fat diet show elevated glucagon levels and decreased secretion of somatostatin, a potent inhibitor of insulin and glucagon release, revealing that intra-islet somatostatin signaling may also play a role in FFA-mediated glucagon increases [18,19]. Others observed that mice fed a high-fat diet demonstrate increased alpha cell mass [20]. In rats, on the contrary, a high-fat diet reduced glucagon levels or had no effect [21]. In healthy lean men, neither oral nor intravenous administration of a lipid emulsion led to significant glucagon level alterations, although glucagon tended to increase during the first 30 min after oral intake [22]. In contrast, the ingestion of oleic acid caused modest acute stimulation of glucagon secretion [23]. A study comparing fatty acids of different chain lengths in meal challenge tests observed the greatest increases in glucagon in response to olive oil (containing long-chain fatty acids), followed by C-8 dietary oil (digested to medium-chain fatty acids), but no change in response to tributyrin (containing short-chain fatty acids) was observed [24], thus confirming that the acute glucagonotropic effect of lipids may depend on fat composition. Heparin-induced elevation of FFAs decreased glucagon secretion, while nicotinic acid-induced suppression of FFAs elevated glucagon secretion in humans [25]. Despite some contrary findings, it appears that alongside the predominant roles of glucose and amino acids, lipids may also influence the physiological secretion of glucagon. To our knowledge, no long-term effects of high-fat diets on glucagon levels have been reported in healthy subjects.

Given the underexplored role of dietary fat intake on glucagon levels in humans, we investigated the response of glucagon in 92 lean, healthy adult twins to low and high fat intake for 6 weeks each. We moreover tested the response to a further 6 weeks of high protein, low fat intake in 24 participants who agreed to continue the study. We assessed the interaction with insulin secretion, circulating free fatty acids, amino acids and hepatic fat content.

## 2. Materials and Methods

### 2.1. Study Protocol and Participants

**Figure 1:**
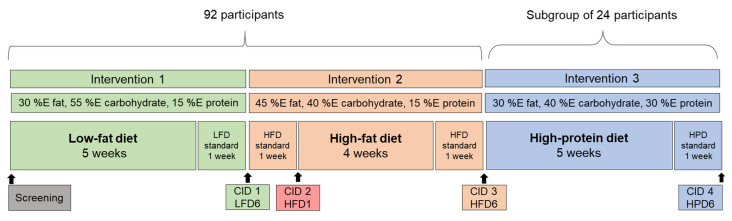
The design of the study. Following initial screening, the 92 subjects underwent two 6-week interventions: first, they consumed a low-fat, high-carbohydrate diet (LFD), which was carried out to standardize the diet of the subjects, and second, the participants consumed an isocaloric high-fat diet (HFD). A total of 24 of the subjects additionally participated in a third 6-week intervention, a high-protein diet (HPD). Clinical investigation days (CIDs) took place at the end of each intervention and after one week of the HFD to examine possible short-term effects. A standardized diet was provided one week prior to each CID to increase each participant’s compliance.

The NutriGenomic Analysis in Twins (NUGAT) study protocol was approved by the ethics committee of the Charité-Universitätsmedizin Berlin (EA4/021/09, date of approval 26 September 2009) and conducted in accordance with the principles of the Helsinki Declaration of 1975, as revised in 2000. Prior to the study, all participants gave written informed consent. The NUGAT study was registered at ClinicalTrials.gov: NCT01631123. Details of recruitment and enrollment of the study participants, the initial screening visit and the exclusion criteria have been published elsewhere [26]. Since genetic variance analyses were the primary endpoint of this study, only twins were included and a randomized controlled design was deliberately avoided. A total of 92 subjects (46 pairs of twins—34 monozygotic and 12 dizygotic), including 58 females and 34 males, age 18 to 70 years and with a body mass index (BMI) from 18 to 29 kg/m^2^ and a BMI difference <3 kg/m^2^ between twins completed the study between September 2009 and September 2012. To standardize food intake, the study participants were first asked to consume a healthy diet for 6 weeks, with 30% of their energy (%E) coming from fats, specifically one-third each for saturated fatty acids (SFAs), mono-unsaturated fatty acids (MUFAs) and poly-unsaturated fatty acids (PUFAs). The protein content was 15 %E and carbohydrates were 55 %E, corresponding to the low-fat recommendations of the German Society of Nutrition at that time. To increase compliance and ensure a standardized dietary pattern, approximately 70% of food was supplied together with detailed meal plans for the last week before the first clinical investigation day (CID1). The participants were then switched to a high-fat diet (HFD) for 6 weeks containing 45 %E of fat (18% SFAs, 17% MUFAs and 10% PUFAs), 40 %E of carbohydrates and 15 %E of protein. Clinical investigations were performed after 1 week of the HFD (CID2), in which food was mainly supplied, and after another 5 weeks of the HFD (CID3), again with most foods eaten during the week before CID3 supplied. Thereafter, 24 twins continued the study with the intake of a low-fat (30 %E) but high-protein (30 %E) and moderate-carbohydrate (40 %E) diet for 6 weeks until CID4 (Figure 1). On each CID, anthropometric measurements were performed. Additionally, magnetic resonance spectroscopy was performed using a Magnetom Avanto 1.5T whole-body scanner (Siemens Healthcare, Erlangen, Germany) for quantification of liver fat content (intrahepatic lipids) on CID1, CID2 and CID3 [27]. All three diets were isocaloric to avoid significant changes in body weight which may affect glucagon levels independently from food composition. To obtain homogenous food intakes, all participants received detailed dietary advice and meal plans from an experienced dietician. The subjects completed 6 dietary records of 5–6 days at specified times during the study, as published previously [26], and the HPD subgroup completed an additional food record during the high-protein intervention period.

### 2.2. Blood Parameters

On each CID, blood samples were drawn in the fasted state (>10 h since last food intake) in the morning from the forearm vein and centrifuged at 1800× *g* for 10 min at 4 °C, and serum was stored at −80 °C until analysis. The determination of routine serum parameters (e.g., total cholesterol, triglycerides, free fatty acids) was performed using an automated analyzer (ABX Pentra 4000; ABX, Montpellier, France). LDL cholesterol concentrations were calculated using the Friedewald equation. Fasting levels of the following amino acids were measured in EDTA plasma samples of CID1-4 via liquid chromatography–mass spectrometry (LC–MS): alanine, arginine, asparagine, aspartic acid, citrulline, cystine, glutamine, glutamic acid, glycine, histidine, leucine, lysine, methionine, ornithine, phenylalanine, proline, serine, threonine, tryptophan, tyrosine, and valine. Isoleucine was not determined and is consequently not reported here. Glucagon and insulin were measured in the fasting serum of all participants on each CID with specific human ELISA kits (Mercodia, Uppsala, Sweden). In addition, on CID 1, 2 and 3, a subgroup of 14 participants randomly taken from the entire study group consumed a test meal (Fresubin^®^ Energy drink, Fresenius Kabi, Bad Homburg, Germany) at noon, and we measured glucagon serum levels before and 240 min after test meal consumption. 

The homeostasis model assessment–estimated insulin resistance (HOMA-IR) was calculated as fasting insulin [mU/L] multiplied by fasting glucose [mmol/L] divided by 22.5 [28].

### 2.3. Heritability

Heritability was estimated by applying the “ACE structural equation model” for every CID. This model analyzes covariance based on comparing the degree of concordance in the form of the correlation coefficient within and between monozygous versus dizygous twin pairs. The proportion of variance is partitioned into (A) additive genetic influences, (C) common environmental influences and (E) individual environmental influences.

### 2.4. Statistical Analysis

Prior to data analysis, a test of plausibility was performed and unusual values that were outside of the 3-fold interquartile range were declared as extreme outliers and were not considered in further analysis. The Shapiro–Wilk test was used to assess variables for normal distribution. Mean values for continuous data were compared using repeated-measures ANOVA followed by the Bonferroni adjusted post hoc test. The requirements for the ANOVA were tested by the Shapiro–Wilk test and Mauchly’s sphericity test with ln- and/or Greenhouse–Geisser transformation if necessary. The Friedman test as a nonparametric equivalent of the ANOVA was used to verify significant results for non-normally distributed data. 

To analyze whether the age of the subjects had an impact on high-fat-diet-induced glucagon changes, we split the cohort into 3 age subgroups (tertiles: 18–23 years, 24–30 years, 31–70 years) and conducted Friedman tests followed by Bonferroni adjusted post hoc tests for each age group again. A nonparametric one-way ANOVA (Kruskal–Wallis test) was applied for the comparison between the age tertiles. To test for sex differences, we split the cohort by sex and the Friedman test with the Bonferroni adjusted post hoc test was applied for males and females separately. The Mann–Whitney U test was used to check for significant differences between glucagon levels in females and males. 

Pearson’s and Spearman’s rank correlation coefficients were used for correlation analysis of variables with normal and skewed distribution, respectively. In cases of missing data, pairwise deletion was applied. Statistical analyses were processed using SPSS 28.0 (SPSS Inc., Chicago, IL, USA), and *p* < 0.05 was considered significant. Values are expressed as the mean ± SEM, unless otherwise stated. The graphs were generated with GraphPad Prism 9 (GraphPad, San Diego, CA, USA).

## 3. Results

### 3.1. Participants’ Characteristics and Compliance

We analyzed 92 healthy participants whose baseline anthropometric and clinical characteristics are shown in Table 1. The macronutrient intake, calculated from dietary records with the PRODI^®^4.5 software (Nutri-Science GmbH, Freiburg, Germany), was 35 %E fat, 50 %E carbohydrates and 15 %E protein prior to the study, 29 %E fat, 55 %E carbohydrates and 16 %E protein during the LFD, 44 %E fat, 42 %E carbohydrates and 14 %E protein during the HFD, and 29 %E fat, 40 %E carbohydrates and 31 %E protein during the HPD, reflecting high dietary compliance of the participants during all study phases (Table S1). Although the study followed an isocaloric approach, the bodyweight of our subjects slightly decreased in response to the LFD (−0.98 kg, *p* < 0.001), increased again after 6 weeks of the HFD (+0.40 kg, *p* = 0.124 vs. LFD) and increased minutely again with the HPD (+0.12 kg, *p* = 1.00 vs. HFD6). However, these weight changes were within a range of natural fluctuation, and thus, the effects of weight alterations were successfully reduced to a minimum in the present study. 

As expected, we observed a rise in total cholesterol and its LDL and HDL fractions in response to the HFD (Table S2), which again represents the good compliance of the participants to the dietary intervention.

### 3.2. Changes in Serum Glucagon and Metabolic Parameters in Response to High Dietary Fat Intake

**Figure 2:**
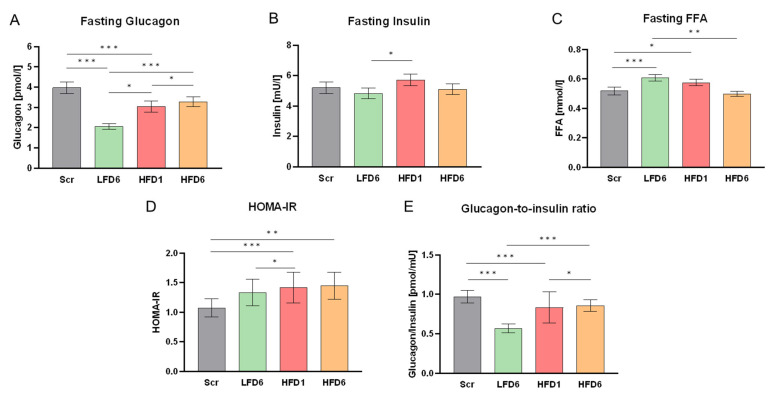
The effects of varying dietary fat intake on glucose metabolism. All parameters are shown for screening (Scr, gray) after a low-fat diet for 6 weeks (LFD6, green), after a high-fat diet for 1 week (HFD1, red) and after a high-fat diet for 6 weeks (HFD6, orange). The values are shown as the mean ± SEM. * *p* < 0.05; ** *p* < 0.01; *** *p* < 0.001. (**A**) Fasting serum glucagon values. Glucagon showed a pronounced decrease in response to 6 weeks of low fat intake and increased significantly after 1 week of high fat intake and even further after 6 weeks. (**B**) Fasting serum insulin. Fasting insulin levels increased slightly but significantly after 1 week of high fat intake, however returned to baseline levels after 6 weeks. (**C**) Fasting serum free fatty acids (FFAs). FFAs changed contrastingly to glucagon levels and increased after the LFD but decreased after the HFD. (**D**) Homeostasis model assessment–estimated insulin resistance. The HOMA-IR increased slightly due to high dietary fat intake; nevertheless, the subjects remained very insulin-sensitive at all time points. (**E**) Glucagon-to-insulin ratio. As insulin levels did not change extensively, the glucagon-to-insulin ratio acts similarly to the fasting glucagon levels, and thus decreased in response to the LFD and increased in response to the HFD.

Fasting glucagon levels increased within 1 week after the onset of the HFD and increased further over the 6 weeks of the HFD to almost double the values after the LFD (Figure 2). To identify factors involved in the increases in glucagon, we first determined correlations with anthropometric parameters and insulin resistance measured as HOMA-IR. The HFD had remarkably modest effects on insulin resistance in this lean and healthy population. Insulin levels increased slightly after 1 week of the HFD and diminished again to baseline levels after 6 weeks of the HFD, while glucose levels remained unchanged, resulting in a small but significant increase in HOMA-IR (Figure 2). However, the marginal increase in HOMA-IR did not correlate with the changes in glucagon levels (Table S4).

We did not observe a significant correlation between intrahepatic lipids and glucagon neither before nor after the HFD (Table S3). It should be noted that the lean and healthy twins had a low intrahepatic lipid content at baseline which did not change in response to the dietary interventions (Table S2). 

The glucagon-to-insulin ratio decreased in response to the LFD and increased in response to the HFD, without changes in glucose (Figure 2). Thus, higher fasting glucagon after the HFD did not elevate fasting blood sugar levels as might be expected, particularly since the changes in insulin and insulin resistance were rather small.

The high-fat diet significantly reduced circulating FFAs in the fasted state over the 6 weeks of the study (Figure 2). Notably, the previous LFD significantly increased FFAs compared to screening values, which might be due to the slightly higher SFA intake (13 %E) of the twins in their habitual diet before the study. The HFD-induced decrease in FFAs might be related to the increase in glucagon. However, we did not find a correlation between the absolute values or the changes in FFAs and glucagon (Tables S3 and S4). 

The sum of all measured circulating amino acids did not change in response to the HFD. Only proline showed a significant increase after one week of high dietary fat intake but decreased again after 6 weeks. None of the other plasma amino acids showed significant changes after 1 week of the HFD. After 6 weeks of the HFD, asparagine slightly decreased, whereas tryptophan and valine increased compared to LFD levels (Table S5). Interestingly, the total level of measured circulating amino acids correlated with the glucagon levels after 6 weeks of the HFD; however, the changes in amino acids and glucagon in response to the HFD did not correlate (Tables S6 and S7).

### 3.3. Impact of Age and Sex on Glucagon Levels

To investigate whether age and sex play a role in affecting glucagon responses to food intake, we conducted an age-stratified and sex-stratified analysis. A comparison of age tertiles showed a glucagon decline after 6 weeks of the LFD and an increase in response to the HFD in all 3 groups independent of age, and no significant differences between the age tertiles were found (Figure S1). Thus, in the present lean population, age appears to have no influence on diet-mediated glucagon changes. In contrast, sex seems to play a role in affecting glucagon levels, as we observed significantly higher glucagon levels in males compared to females at baseline, after 6 weeks of the LFD and after 6 weeks of the HFD. Nevertheless, both sexes showed a significant increase in glucagon levels in response to 6 weeks of high dietary fat intake (Figure S2).

### 3.4. Changes in Postprandial Glucagon in Response to the HFD

In a subgroup of 14 participants randomly taken from the entire study group, we further assessed whether glucagon levels changed in response to a test meal (Fresubin^®^ Energy drink) that was administered at noon after 6 weeks of the LFD, after 1 week of the HFD and after 6 weeks of the HFD. We measured serum glucagon before test meal consumption (0 min) and 240 min after the meal. In this subgroup, glucagon showed a slight but not significant increase before intake of the test meal at noon after 6 weeks of the HFD. However, the levels after the meal increased significantly, suggesting that both fasted and postprandial levels of glucagon increase upon prolonged intake of an HFD in healthy people (Figure S3).

### 3.5. Serum Glucagon and Metabolic Parameter Changes in Response to High Dietary Protein Intake

**Figure 3:**
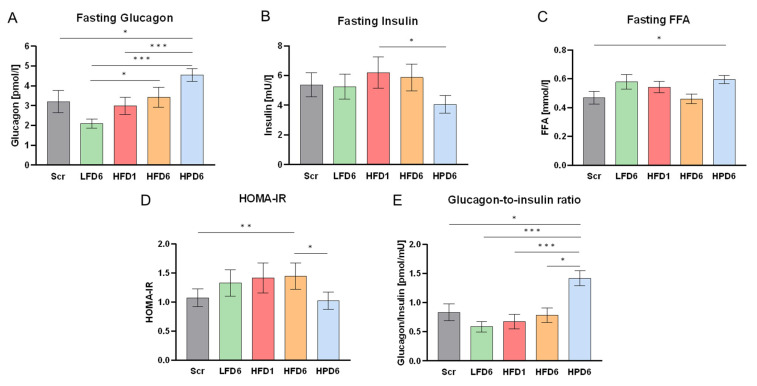
The effects of high protein intake on glucose metabolism. All parameters are shown for screening (Scr, gray), after the low-fat diet for 6 weeks (LFD6, green), after the high-fat diet for 1 week (HFD1, red) and after the high-fat diet for 6 weeks (HFD6, orange) for the subgroup of 24 participants that additionally participated in 6 weeks of a high-protein diet (HPD6, blue). In general, the responses of this subgroup to the LFD and HFD were similar to those of the total cohort (see Figure 2). The values are shown as the mean ± SEM. * *p* < 0.05; ** *p* < 0.01; *** *p* < 0.001. (**A**) Fasting serum glucagon values. Glucagon showed a pronounced increase after 6 weeks of the HPD. (**B**) Fasting serum insulin. Fasting insulin levels decreased significantly after 6 weeks of high protein intake. (**C**) Fasting serum free fatty acids (FFAs). FFAs were highest in response to 6 weeks of the HPD. (**D**) Homeostasis model assessment–estimated insulin resistance. HOMA-IR decreased slightly but significantly during the HPD. (**E**) Glucagon-to-insulin ratio. The glucagon-to-insulin ratio increased most intensively with the HPD.

The further switch to a low-fat, high-protein diet was studied in a subgroup of 24 participants who agreed to another 6 weeks of controlled dietary intake. This subgroup was, on average, 8 years older and had a slightly higher BMI of 24 ± 2 kg/m^2^, which is still in the range of normal weight (Table 1). The responses of this subgroup to the LFD and HFD were similar to those of the 92 twins (Figure 3), with a pronounced decrease in glucagon under the LFD compared to screening and an increase during the HFD. The HPD further increased glucagon values compared to the HFD, despite the lower fat content. The FFA profile (Figure 3) and HDL cholesterol levels were similar to those under the low-fat diet, confirming the adherence to lower fat intake (Table S8). This suggests that the HPD further increased fasting glucagon values independently of the effects of fat.

Insulin levels were significantly reduced under the HPD, resulting in a pronounced increase (more than 2-fold compared to after the LFD) of the glucagon-to-insulin ratio (Figure 3). Nevertheless, glucose levels remained unchanged, which resulted in an improvement in insulin sensitivity, as calculated by HOMA-IR values. The high glucagon levels thus did not impair glucose metabolism during the 6 weeks of the high-protein intervention.

The total concentration of amino acids that was measured in this study did not alter with the HPD. Nevertheless, significant changes in response to the HPD occurred in several amino acids, but as some decreased (alanine, glutamine, glycine, proline) and others increased (glutamic acid, leucine, lysine, methionine, phenylalanine, threonine, tryptophan, valine), no change in the overall level was observed. Except for glutamic acid, all amino acids that increased in response to the HPD were essential amino acids (Table S9). Surprisingly, we found less correlations between amino acids and glucagon with the HPD compared to the LFD and HFD, as only serine correlated with glucagon after the HPD (Table S6). Instead, the change in total amino acids in response to the HPD was negatively correlated with the change in glucagon (Table S7).

### 3.6. Heritability of Glucagon Levels

The twin design allowed for the calculation of the heritability of the glucagon levels at screening and in the presence of the different diets (Figure S4). The glucagon serum levels at screening were not heritable, despite a slight but significant correlation among monozygotic twin pairs (r = 0.353, *p* = 0.041). After 6 weeks of the low-fat diet, the significant correlation of glucagon levels (r = 0.534, *p* = 0.001) among the monozygotic twins was more pronounced, but no correlation was observed among the dizygotic twins (r = 0.308, ns), resulting in an estimated heritability of 45%. Remarkably, this heritability and the significant correlation among monozygotic twin pairs was lost after 6 weeks of the HFD, suggesting that non-heritable factors play a pronounced role in this condition. In addition, the change in glucagon levels (delta glucagon) in response to altered nutrition was not heritable at any time of the study. The high-protein diet included 10 monozygotic and 2 dizygotic pairs of twins and thus did not allow for the calculation of heritability. Nevertheless, the glucagon levels showed a high correlation in the monozygotic pairs (r = 0.733, *p* = 0.025) after 6 weeks of high protein intake.

## 4. Discussion

We report extensive diet-dependent changes in serum glucagon in lean healthy young subjects. Glucagon showed a pronounced decrease in response to the LFD. In contrast, glucagon progressively increased after 1 and 6 weeks of the HFD, which has not been reported before. The increased fat intake was in exchange for carbohydrates, which were reduced from 55 to 40 %E. This corresponds to a moderate restriction of carbohydrates which is unlikely to increase glucagon levels extensively. Previous studies showed low carbohydrate intake to significantly increase glucagon levels [29,30,31]; however, these trials used considerably lower carbohydrate portions than in our present study. We hypothesize that the observed rise in glucagon in response to the HFD is not solely due to the moderate carbohydrate restriction, but to its combination with higher nutritional fat intake. The ingestion of fat alone has previously been shown to modestly increase prandial glucagon release in healthy subjects and subjects with type 2 diabetes [32]. However, the ingestion of solely protein was found to increase glucagon to a higher extent than fat [23,32]. These results are in line with our data which show the highest fasting glucagon levels in response to 6 weeks of the HPD, reflecting the potent stimulation of glucagon secretion through amino acids that has been reported since the 1960s [7,33]. Taken together, this supports the hypothesis that fat intake plays a role in glucagon secretion, even though it appears to not be as pertinent as protein/amino acid intake to stimulating glucagon release. 

The total fasting blood amino acid concentration did not alter with the high protein intake in our study, and we found a negative correlation between the change in glucagon and the change in the total amino acids in response to 6 weeks of the HPD. This may appear contradictory at first, but appears reasonable under consideration of the liver–alpha-cell axis: postprandial amino acids function as glucagon secretagogues, causing a rise in glucagon which in reverse leads to elevated amino acid degradation by the urea cycle in the liver, thereby normalizing the circulating amino acid concentrations [7]. A previous study in healthy men showed lower fasting blood amino acids in a high-protein-intake group compared to a normal-protein-intake group, except for leucine, methionine and tyrosine [34]. In line with these findings, leucine and methionine levels, among others, were significantly elevated after the HPD in our cohort, whereas alanine, glutamine, glycine and proline concentrations diminished after the HPD. Fernstrom et al. have also reported lower fasting glycine and alanine plasma concentrations in subjects that consumed 150 g egg protein/day compared to those consuming 75 or 0 g protein daily for 5 days [35]. In contrast to our findings, Forslund et al. reported fasting lysine, phenylalanine, threonine, tryptophan and valine to be lower in the high-protein group [34], whereas the fasting levels of these essential amino acids increased significantly after the HPD in our study. Notably, the increase in branched chain amino acids may be explained by the lowered insulin levels which regulate branched chain amino acid degradation [36,37]. Several studies did not show an upregulation of branched chain amino acids with higher intakes [38,39]. These differences in the amino acid responses may be due to the varying nutritional intake of the specific amino acids and the different study durations. However, besides the amount and composition of amino acids supplied, several tissues and metabolic pathways impact the concentrations of circulating plasma amino acids, making it very difficult to identify the complete metabolic background of the observed amino acid alterations. A drop in fasting glucogenic amino acids in response to high nutritional protein consumption has been described in rats and related to their sustained catabolism [40]. In addition to increased amino acid catabolism and renal clearance, decreased de novo synthesis and restrained body protein breakdown have been postulated as responsible for the inverse response of glutamine and alanine concentrations to high protein intake in humans [41,42]. Interestingly, glutamine, alanine, glycine and proline have been described as potent glucagon secretagogues [7,43,44]. Moreover, alanine and proline were found to be involved in the acute regulation of the liver–alpha-cell axis in female mice [44], which may play a role in their significant decrease after 6 weeks of an HPD.

Studies comparing high-fat vs. low-fat meals confirmed greater acute stimulation of glucagon by the high-fat meal in healthy [45,46] and overweight subjects [47]. Raben and coworkers [48] subjected 10 healthy lean and 8 normal-weight post-obese women to high-fat, high-starch or high-sucrose diets for 2 weeks in a crossover design and did not find significant changes in fasting or postprandial glucagon levels, although fasting levels of glucagon increased by 33%. The participants consumed low-fat diets for 2 weeks containing 28 %E of fat prior to the HFD with 46 %E of fat. These results are compatible with our data, which show a time-dependent further increase in glucagon levels over the 6 weeks. This suggests a delayed metabolic adaptation similar to the alterations in LDL and HDL cholesterol. The mechanisms involved are unclear. 

The role of glucagon in lipid metabolism has been established by the increases in liver fat induced by glucagon antagonists in human trials [12,13], which most likely relates to the induction of hepatic lipolysis and lipid oxidation by glucagon [14,15]. Thus, the elevation of glucagon in the present study might be interpreted as a metabolic response to prevent hepatic fat accumulation upon high dietary fat intake. In line with that, the circulating FFAs decreased progressively with the HFD, which could result from their increased hepatic oxidation and a decrease in hepatic lipogenesis through elevated glucagon [49]. Moreover, this may explain the absence of increases in liver fat by the HFD in our healthy subjects, which contrasts previous reports on obese subjects [50]. The diet-induced changes in glucagon found in the present study are also contrary to observations in diabetes patients who did not show decreases in glucagon upon intake of a low-fat diet containing 30 %E of fat compared to 42 %E before the start of the intervention or increases in response to a high-protein diet for 6 weeks. Notably, plasma amino acid levels did not change in this study [39,51]. However, chronic hyperglycemia is associated with profound alterations in glucagon secretion, which may account for the differences [52]. 

Glucagon levels were shown to be correlated with hepatic fat content in obese individuals with [51] and without type 2 diabetes [53]. This correlation was not observed in our healthy population who had low levels of liver fat content and included only seven individuals with intrahepatic lipids above the threshold of 5.56% defining non-alcoholic fatty liver disease [54]. Moreover, the lean and healthy twins were resistant to high-fat-diet-induced increases in liver fat over the 6 weeks of the isocaloric study.

A comparison of glucagon levels between male and female participants revealed that despite similar glucagon responses to the LFD and HFD in both sexes, male participants showed constantly higher fasting glucagon levels. Matching our results, a recently published study in individuals with type 2 diabetes found higher fasting and postprandial glucagon levels in men compared to women [55]. Considered together with similar results from oral glucose tolerance tests in healthy subjects [56], it emerges that sex plays a role in glucagon metabolism. The involvement of estrogen in the regulation of glucagon secretion has been suggested [57]; however, future research is needed to completely understand the mechanisms underlying this sex difference.

The glucagon-to-insulin ratio increased in response to 6 weeks of the HFD and even further after 6 weeks of the HPD. Remarkably, these distinct increases occurred without changes in blood glucose levels. This indicates that healthy people may desensitize to the glucose-producing effects of glucagon or counteract hepatic glucose production via unclear mechanisms upon permanently elevated glucagon levels. Capozzi and coworkers [58] have already reported that under prandial conditions, glucagon functions more as an insulinotropic agent ensuring euglycemia rather than further elevating blood glucose levels via hepatic glucose production.

The major strengths of the current study are the isocaloric approach, enabling results independent from presumably confounding bodyweight changes, and the high dietary compliance of the subjects. The limitations of the study include the moderate number of subjects analyzed and the restriction to Caucasian ethnicity; therefore, its conclusions may not apply to other populations because of ethnic or genetic differences. Additionally, the study was not randomized and targeted similar dietary conditions for all twins using the differences in pairwise responses to address the inheritance in dietary changes. Moreover, we only measured total serum FFAs without further differentiation, though the ability of FFAs to stimulate glucagon secretion may depend on fatty acid length and the degree of saturation [13,24].

## 5. Conclusions

In conclusion, a high-fat, low-carbohydrate diet strongly raises glucagon levels and the glucagon-to-insulin ratio in healthy lean subjects in comparison to an isocaloric low-fat, high-carbohydrate diet. Interestingly, blood glucose levels, liver fat and insulin resistance showed no clinically relevant increases. Thus, the observed rise in glucagon might be interpreted as a metabolic response to high dietary fat intake to prevent hepatic fat accumulation, as increases in glucagon have been previously shown to induce hepatic lipolysis and fat oxidation [14,15]. This allows for an alternative perspective to position elevated glucagon secretion observed in type 2 diabetes as a mechanism to compensate, rather than induce, dysregulated homeostasis. 

## Figures and Tables

**Table 1:** The baseline clinical parameters of all subjects and the subgroup that additionally participated in the high-protein intervention.

Baseline Characteristics (Mean ± SD)	All Participants n = 92	HPD Subgroup n = 24
Sex [female/male]	58/34	10/14
Zygosity [mono/di]	68/24	20/4
Age [y]	31 ± 14	39 ± 15
BMI [kg/m^2^]	23 ± 3	24 ± 2
WHR	0.81 ± 0.07	0.84 ± 0.06
Systolic BP [mm Hg]	118 ± 13	117 ± 11
Diastolic BP [mm Hg]	74 ± 9	76 ± 6
Fasting insulin [mU/L]	5.21 ± 3.68	5.38 ± 4.00
Fasting glucose [mmol/L]	4.78 ± 0.48	5.05 ± 0.46
HbA1c [%]	5.0 ± 0.4	5.0 ± 0.4
HbA1c [mmol/mol]	31 ± 5	31 ± 5
Fasting glucagon [pmol/L]	3.97 ± 2.71	3.21 ± 2.75
Fasting total cholesterol [mmol/L]	4.58 ± 0.93	4.69 ± 0.98
Fasting HDL cholesterol [mmol/L]	1.38 ± 0.35	1.32 ± 0.34
Fasting LDL cholesterol [mmol/L]	2.73 ± 0.77	2.89 ± 0.83
Fasting triglycerides [mmol/L]	0.99 ± 0.44	0.94 ± 0.38
Fasting FFA [mmol/L]	0.52 ± 0.26	0.47 ± 0.22

Values are shown as mean ± SD. BP, blood pressure; FFA, free fatty acids; HPD, high-protein diet; WHR, waist-to-hip ratio.

## Data Availability

The data are available on request due to privacy restrictions.

## Supplementary Materials

The following supporting information can be downloaded at: https://www.mdpi.com/article/10.3390/nu16223905/s1, Figure S1: Glucagon changes in age subgroups. Figure S2: Glucagon changes grouped by sex. Figure S3: Glucagon levels before and after test meal. Figure S4: Heritability of glucagon. Table S1: Target and actual macronutrient composition for each intervention phase. Table S2: Characteristics of all participants (n = 92) in response to high-fat diet. Table S3: Correlation analysis of clinical parameters with fasting glucagon. Table S4: Correlation analysis of the change (delta %) in fasting glucagon with the change in clinical parameters. Table S5: Changes in amino acids of all participants in response to high-fat diet. Table S6: Correlation analysis of fasting circulating amino acids with fasting glucagon. Table S7: Correlation analysis of the change (delta) in fasting glucagon with the change in fasting circulating amino acids. Table S8: The characteristics of the subgroup of participants that continued with the high-protein intervention (n = 24) measured on each clinical investigation day. Table S9: Changes in fasting amino acids in the subgroup of participants that continued with the high-protein intervention (n = 24).

## References

[1] Rohrer S., Menge B.A., Gruber L., Deacon C.F., Schmidt W.E., Veldhuis J.D., Holst J.J., Meier J.J. (2012). Impaired crosstalk between pulsatile insulin and glucagon secretion in prediabetic individuals. J. Clin. Endocrinol. Metab..

[2] Menge B.A., Gruber L., Jorgensen S.M., Deacon C.F., Schmidt W.E., Veldhuis J.D., Holst J.J., Meier J.J. (2011). Loss of inverse relationship between pulsatile insulin and glucagon secretion in patients with type 2 diabetes. Diabetes.

[3] Meier J.J., Kjems L.L., Veldhuis J.D., Lefebvre P., Butler P.C. (2006). Postprandial suppression of glucagon secretion depends on intact pulsatile insulin secretion: Further evidence for the intraislet insulin hypothesis. Diabetes.

[4] Moede T., Leibiger I.B., Berggren P.O. (2020). Alpha cell regulation of beta cell function. Diabetologia.

[5] Zhu L., Dattaroy D., Pham J., Wang L., Barella L.F., Cui Y., Wilkins K.J., Roth B.L., Hochgeschwender U., Matschinsky F.M. (2019). Intra-islet glucagon signaling is critical for maintaining glucose homeostasis. JCI Insight.

[6] Meier J.J., Deacon C.F., Schmidt W.E., Holst J.J., Nauck M.A. (2007). Suppression of glucagon secretion is lower after oral glucose administration than during intravenous glucose administration in human subjects. Diabetologia.

[7] Dean E.D. (2020). A Primary Role for alpha-Cells as Amino Acid Sensors. Diabetes.

[8] Dean E.D., Li M., Prasad N., Wisniewski S.N., Von Deylen A., Spaeth J., Maddison L., Botros A., Sedgeman L.R., Bozadjieva N. (2017). Interrupted Glucagon Signaling Reveals Hepatic alpha Cell Axis and Role for L-Glutamine in alpha Cell Proliferation. Cell Metab..

[9] Okun J.G., Rusu P.M., Chan A.Y., Wu Y., Yap Y.W., Sharkie T., Schumacher J., Schmidt K.V., Roberts-Thomson K.M., Russell R.D. (2021). Liver alanine catabolism promotes skeletal muscle atrophy and hyperglycaemia in type 2 diabetes. Nat. Metab..

[10] Gerich J.E., Charles M.A., Grodsky G.M. (1974). Characterization of the effects of arginine and glucose on glucagon and insulin release from the perfused rat pancreas. J. Clin. Investig..

[11] Wewer Albrechtsen N.J., Pedersen J., Galsgaard K.D., Winther-Sorensen M., Suppli M.P., Janah L., Gromada J., Vilstrup H., Knop F.K., Holst J.J. (2019). The Liver-alpha-Cell Axis and Type 2 Diabetes. Endocr. Rev..

[12] Scheen A.J., Paquot N., Lefebvre P.J. (2017). Investigational glucagon receptor antagonists in Phase I and II clinical trials for diabetes. Expert Opin. Investig. Drugs.

[13] Galsgaard K.D., Pedersen J., Knop F.K., Holst J.J., Wewer Albrechtsen N.J. (2019). Glucagon Receptor Signaling and Lipid Metabolism. Front. Physiol..

[14] Perry R.J., Zhang D., Guerra M.T., Brill A.L., Goedeke L., Nasiri A.R., Rabin-Court A., Wang Y., Peng L., Dufour S. (2020). Glucagon stimulates gluconeogenesis by INSP3R1-mediated hepatic lipolysis. Nature.

[15] Petersen K.F., Dufour S., Mehal W.Z., Shulman G.I. (2024). Glucagon promotes increased hepatic mitochondrial oxidation and pyruvate carboxylase flux in humans with fatty liver disease. Cell Metab..

[16] Briant L.J.B., Dodd M.S., Chibalina M.V., Rorsman N.J.G., Johnson P.R.V., Carmeliet P., Rorsman P., Knudsen J.G. (2018). CPT1a-Dependent Long-Chain Fatty Acid Oxidation Contributes to Maintaining Glucagon Secretion from Pancreatic Islets. Cell Rep..

[17] Armour S.L., Frueh A., Chibalina M.V., Dou H., Argemi-Muntadas L., Hamilton A., Katzilieris-Petras G., Carmeliet P., Davies B., Moritz T. (2023). Glucose Controls Glucagon Secretion by Regulating Fatty Acid Oxidation in Pancreatic α-Cells. Diabetes.

[18] Gromada J., Franklin I., Wollheim C.B. (2007). α-Cells of the Endocrine Pancreas: 35 Years of Research but the Enigma Remains. Endocr. Rev..

[19] Kellard J.A., Rorsman N.J.G., Hill T.G., Armour S.L., van de Bunt M., Rorsman P., Knudsen J.G., Briant L.J.B. (2020). Reduced somatostatin signalling leads to hypersecretion of glucagon in mice fed a high-fat diet. Mol. Metab..

[20] Ellingsgaard H., Ehses J.A., Hammar E.B., Van Lommel L., Quintens R., Martens G., Kerr-Conte J., Pattou F., Berney T., Pipeleers D. (2008). Interleukin-6 regulates pancreatic α-cell mass expansion. Proc. Natl. Acad. Sci. USA.

[21] Eisenstein A.B., Strack I., Steiner A. (1974). Increased hepatic gluconeogenesis without a rise of glucagon secretion in rats fed a high fat diet. Diabetes.

[22] Lindgren O., Carr R.D., Deacon C.F., Holst J.J., Pacini G., Mari A., Ahrén B. (2011). Incretin Hormone and Insulin Responses to Oral Versus Intravenous Lipid Administration in Humans. J. Clin. Endocrinol. Metab..

[23] Carr R.D., Larsen M.O., Winzell M.S., Jelic K., Lindgren O., Deacon C.F., Ahren B. (2008). Incretin and islet hormonal responses to fat and protein ingestion in healthy men. Am. J. Physiol. Endocrinol. Metab..

[24] Mandøe M.J., Hansen K.B., Hartmann B., Rehfeld J.F., Holst J.J., Hansen H.S. (2015). The 2-monoacylglycerol moiety of dietary fat appears to be responsible for the fat-induced release of GLP-1 in humans. Am. J. Clin. Nutr..

[25] Gerich J.E., Langlois M., Schneider V., Karam J.H., Noacco C. (1974). Effects of alternations of plasma free fatty acid levels on pancreatic glucagon secretion in man. J. Clin. Investig..

[26] Schuler R., Osterhoff M.A., Frahnow T., Seltmann A.C., Busjahn A., Kabisch S., Xu L., Mosig A.S., Spranger J., Mohlig M. (2017). High-Saturated-Fat Diet Increases Circulating Angiotensin-Converting Enzyme, Which Is Enhanced by the rs4343 Polymorphism Defining Persons at Risk of Nutrient-Dependent Increases of Blood Pressure. J. Am. Heart Assoc..

[27] Machann J., Thamer C., Schnoedt B., Stefan N., Haring H.U., Claussen C.D., Fritsche A., Schick F. (2006). Hepatic lipid accumulation in healthy subjects: A comparative study using spectral fat-selective MRI and volume-localized 1H-MR spectroscopy. Magn. Reson. Med..

[28] Wallace T.M., Levy J.C., Matthews D.R. (2004). Use and Abuse of HOMA Modeling. Diabetes Care.

[29] Gannon M.C., Nuttall F.Q. (2004). Effect of a high-protein, low-carbohydrate diet on blood glucose control in people with type 2 diabetes. Diabetes.

[30] Fujita Y., Gotto A.M., Unger R.M. (1975). Basal and Postprotein Insulin and Glucagon Levels During a High and Low Carbohydrate Intake and Their Relationships to Plasma Triglycerides. Diabetes.

[31] Shimy K.J., Feldman H.A., Klein G.L., Bielak L., Ebbeling C.B., Ludwig D.S. (2020). Effects of Dietary Carbohydrate Content on Circulating Metabolic Fuel Availability in the Postprandial State. J. Endocr. Soc..

[32] Alsalim W., Tura A., Pacini G., Omar B., Bizzotto R., Mari A., Ahrén B. (2016). Mixed meal ingestion diminishes glucose excursion in comparison with glucose ingestion via several adaptive mechanisms in people with and without type 2 diabetes. Diabetes Obes. Metab..

[33] Ohneda A., Parada E., Eisentraut A.M., Unger R.H. (1968). Characterization of response of circulating glucagon to intraduodenal and intravenous administration of amino acids. J. Clin. Investig..

[34] Forslund A.H., Hambræus L., van Beurden H., Holmbäck U., El-Khoury A.E., Hjorth G., Olsson R., Stridsberg M., Wide L., Åkerfeldt T. (2000). Inverse relationship between protein intake and plasma free amino acids in healthy men at physical exercise. Am. J. Physiol.-Endocrinol. Metab..

[35] Fernstrom J.D., Wurtman R.J., Hammarstrom-Wiklund B., Rand W.M., Munro H.N., Davidson C.S. (1979). Diurnal variations in plasma concentrations of tryptophan, tryosine, and other neutral amino acids: Effect of dietary protein intake. Am. J. Clin. Nutr..

[36] Nie C., He T., Zhang W., Zhang G., Ma X. (2018). Branched Chain Amino Acids: Beyond Nutrition Metabolism. Int. J. Mol. Sci..

[37] Vanweert F., Schrauwen P., Phielix E. (2022). Role of branched-chain amino acid metabolism in the pathogenesis of obesity and type 2 diabetes-related metabolic disturbances BCAA metabolism in type 2 diabetes. Nutr. Diabetes.

[38] Prodhan U.K., Milan A.M., Thorstensen E.B., Barnett M.P.G., Stewart R.A.H., Benatar J.R., Cameron-Smith D. (2018). Altered Dairy Protein Intake Does Not Alter Circulatory Branched Chain Amino Acids in Healthy Adults: A Randomized Controlled Trial. Nutrients.

[39] Markova M., Hornemann S., Sucher S., Wegner K., Pivovarova O., Rudovich N., Thomann R., Schneeweiss R., Rohn S., Pfeiffer A.F.H. (2018). Rate of appearance of amino acids after a meal regulates insulin and glucagon secretion in patients with type 2 diabetes: A randomized clinical trial. Am. J. Clin. Nutr..

[40] Moundras C., Remesy C., Demigne C. (1993). Dietary protein paradox: Decrease of amino acid availability induced by high-protein diets. Am. J. Physiol.-Gastrointest. Liver Physiol..

[41] Matthews D.E., Campbell R.G. (1992). The effect of dietary protein intake on glutamine and glutamate nitrogen metabolism in humans. Am. J. Clin. Nutr..

[42] Yang R.D., Matthews D.E., Bier D.M., Wen Z.M., Young V.R. (1986). Response of alanine metabolism in humans to manipulation of dietary protein and energy intakes. Am. J. Physiol..

[43] Müller W.A., Faloona G.R., Unger R.H. (1971). The effect of alanine on glucagon secretion. J. Clin. Investig..

[44] Galsgaard K.D., Jepsen S.L., Kjeldsen S.A.S., Pedersen J., Wewer Albrechtsen N.J., Holst J.J. (2020). Alanine, arginine, cysteine, and proline, but not glutamine, are substrates for, and acute mediators of, the liver-α-cell axis in female mice. Am. J. Physiol.-Endocrinol. Metab..

[45] Dandona P., Ghanim H., Abuaysheh S., Green K., Batra M., Dhindsa S., Makdissi A., Patel R., Chaudhuri A. (2015). Decreased insulin secretion and incretin concentrations and increased glucagon concentrations after a high-fat meal when compared with a high-fruit and -fiber meal. Am. J. Physiol. Endocrinol. Metab..

[46] Radulescu A., Gannon M.C., Nuttall F.Q. (2010). The Effect on Glucagon, Glucagon-Like Peptide-1, Total and Acyl-Ghrelin of Dietary Fats Ingested with and without Potato. J. Clin. Endocrinol. Metab..

[47] Sloth B., Due A., Larsen T.M., Holst J.J., Heding A., Astrup A. (2009). The effect of a high-MUFA, low-glycaemic index diet and a low-fat diet on appetite and glucose metabolism during a 6-month weight maintenance period. Br. J. Nutr..

[48] Raben A., Holst J.J., Madsen J., Astrup A. (2001). Diurnal metabolic profiles after 14 d of an ad libitum high-starch, high-sucrose, or high-fat diet in normal-weight never-obese and postobese women. Am. J. Clin. Nutr..

[49] Hædersdal S., Andersen A., Knop F.K., Vilsbøll T. (2023). Revisiting the role of glucagon in health, diabetes mellitus and other metabolic diseases. Nat. Rev. Endocrinol..

[50] Luukkonen P.K., Sädevirta S., Zhou Y., Kayser B., Ali A., Ahonen L., Lallukka S., Pelloux V., Gaggini M., Jian C. (2018). Saturated Fat Is More Metabolically Harmful for the Human Liver Than Unsaturated Fat or Simple Sugars. Diabetes Care.

[51] Zhang J., Pivovarova-Ramich O., Kabisch S., Markova M., Hornemann S., Sucher S., Rohn S., Machann J., Pfeiffer A.F.H. (2022). High Protein Diets Improve Liver Fat and Insulin Sensitivity by Prandial but Not Fasting Glucagon Secretion in Type 2 Diabetes. Front. Nutr..

[52] Knudsen J.G., Hamilton A., Ramracheya R., Tarasov A.I., Brereton M., Haythorne E., Chibalina M.V., Spegel P., Mulder H., Zhang Q. (2019). Dysregulation of Glucagon Secretion by Hyperglycemia-Induced Sodium-Dependent Reduction of ATP Production. Cell Metab..

[53] Gar C., Haschka S.J., Kern-Matschilles S., Rauch B., Sacco V., Prehn C., Adamski J., Seissler J., Wewer Albrechtsen N.J., Holst J.J. (2021). The liver-alpha cell axis associates with liver fat and insulin resistance: A validation study in women with non-steatotic liver fat levels. Diabetologia.

[54] Szczepaniak L.S., Nurenberg P., Leonard D., Browning J.D., Reingold J.S., Grundy S., Hobbs H.H., Dobbins R.L. (2005). Magnetic resonance spectroscopy to measure hepatic triglyceride content: Prevalence of hepatic steatosis in the general population. Am. J. Physiol. Endocrinol. Metab..

[55] Huang W., Xie C., Jones K.L., Horowitz M., Rayner C.K., Wu T. (2024). Sex differences in the plasma glucagon responses to a high carbohydrate meal and a glucose drink in type 2 diabetes. Diabetes Res. Clin. Pract..

[56] Horie I., Abiru N., Eto M., Sako A., Akeshima J., Nakao T., Nakashima Y., Niri T., Ito A., Nozaki A. (2018). Sex differences in insulin and glucagon responses for glucose homeostasis in young healthy Japanese adults. J. Diabetes Investig..

[57] Handgraaf S., Dusaulcy R., Visentin F., Philippe J., Gosmain Y. (2018). 17-β Estradiol regulates proglucagon-derived peptide secretion in mouse and human α- and L cells. JCI Insight.

[58] Capozzi M.E., Wait J.B., Koech J., Gordon A.N., Coch R.W., Svendsen B., Finan B., D’Alessio D.A., Campbell J.E. (2019). Glucagon lowers glycemia when β cells are active. JCI Insight.

