# Supplementary material for: Alterations in Glucagon Levels and the Glucagon-to-Insulin Ratio in Response to High Dietary Fat or Protein Intake in Healthy Lean Adult Twins: A Post Hoc Analysis
Source: Nutrients. 2024 Nov 15;16(22):3905. doi: 10.3390/nu16223905 (PMC11597242; doi:10.3390/nu16223905)
Supplement: Supplementary file 1 [file nutrients-16-03905-s001.zip › nutrients-3286058-supplementary.pdf]

Supplementary Materials

Table S1: Target and actual macronutrient composition for each intervention phase.

Macronutrient	Low-fat diet		High-fat diet		High-protein diet	
	Target	Actual	Target	Actual	Target	Actual
Fat %E	30	29	45	44	30	29
Carbohydrate %E	55	55	40	42	40	40
Protein %E	15	16	15	14	30	31

%E, %Energy

Table S2: The characteristics of all participants (n=92) in response to high-fat diet.

	Screening	LFD6	HFD1	HFD6
Weight [kg]	67.6 ± 11.8	66.6 ± 11.7[§§§]	66.5 ± 11.6[§§§]	67.0 ± 11.8[§§††]
Glucose [mmol/L]	4.78 ± 0.48	5.22 ± 0.81[§§§]	5.15 ± 0.62[§§§]	5.22 ± 0.61[§§§]
Insulin [mU/L]	5.21 ± 3.68	4.84 ± 3.17	5.70 ± 3.63	5.11 ± 3.52
HOMA-IR	1.07 ± 0.76	1.33 ± 1.08	1.42 ± 1.26[§§§*]	1.45 ± 1.13[§§]
Glucagon [pmol/L]	3.97 ± 2.71	2.06 ± 1.26[§§§]	3.04 ± 2.53[§§§*]	3.28 ± 2.37[***†]
FFA [mmol/L]	0.52 ± 0.26	0.61 ± 0.21[§§§]	0.58 ± 0.21[§]	0.50 ± 0.18[**]
Total cholesterol [mmol/L]	4.58 ± 0.93	4.29 ± 0.85[§§§]	4.47 ± 0.87[**]	4.70 ± 0.91[***††]
LDL [mmol/L]	2.73 ± 0.77	2.59 ± 0.71[§§]	2.71 ± 0.73[**]	2.86 ± 0.79[***]
HDL [mmol/L]	1.38 ± 0.35	1.27 ± 0.33[§§§]	1.32 ± 0.34[***]	1.41 ± 0.37[***†]
Triglycerides [mmol/L]	0.99 ± 0.44	0.95 ± 0.43	0.89 ± 0.35	0.91 ± 0.37
IHL [%]	n.a.	2.1 ± 3.9	2.1 ± 3.9	2.3 ± 3.8

The values are shown as fasting mean ± SD. FFA, free fatty acid; HFD1, investigation day after high-fat diet for 1 week; HFD6, investigation day after high-fat diet for 6 weeks; HOMA-IR, homeostasis model assessment–estimated insulin resistance; IHL, intrahepatic lipid content; LFD6, investigation day after low-fat diet for 6 weeks; n.a., not available.

[§]p < 0.05 vs. screening; [§§]p < 0.01 vs. screening; [§§§]p < 0.001 vs. screening; *p < 0.05 vs. LFD6; **p < 0.01 vs. LFD6; *** p < 0.001 vs. LFD6; [†]p < 0.05 vs. HFD1; [††]p < 0.01 vs. HFD1.

Table S3: Correlation analysis of clinical parameters with fasting glucagon.

	Glucagon				
	Screening	LFD6	HFD1	HFD6	HPD6
Body weight	0.109	0.174	0.102	0.109	0.221
HbA1c	0.107	0.027	0.194	0.000	-0.127
Glucose	-0.121	0.010	**-0.254***	0.071	0.016
Insulin	**0.249***	0.175	0.059	0.177	0.258
HOMA-IR	**0.217***	0.172	0.000	0.181	0.275
Free fatty acids	**-0.241***	-0.003	0.145	-0.094	0.070
Total cholesterol	**-0.214***	-0.101	-0.028	-0.026	-0.040
LDL	-0.057	-0.007	0.040	0.085	-0.118
HDL	**-0.234***	-0.185	-0.144	**-0.242***	0.005
Triglycerides	-0.157	-0.107	**-0.265****	0.047	0.089
IHL	n.a.	0.195	0.148	0.121	n.a.

Shown is the correlation coefficient (Spearman or Pearson according to data distribution). For clinical investigation days, Screening, LFD6, HFD1 and HFD6 correlations were calculated with the data of all 92 participants; for HPD6, correlations were calculated with the data of the 24 participants that continued with the high-protein diet.

HFD1, investigation day after high-fat diet for 1 week; HFD6, investigation day after high-fat diet for 6 weeks; HOMA-IR, homeostasis model assessment–estimated insulin resistance; HPD6, investigation day after high-protein diet for 6 weeks; IHL, intrahepatic lipid content; LFD6, investigation day after low-fat diet for 6 weeks; n.a., not available.

*$p < 0.05$; **$p < 0.01$.

Table S4: Correlation analysis of the change (delta %) in fasting glucagon with the change in clinical parameters.

	Delta % Glucagon				
	Scr – LFD6	LFD6 – HFD1	HFD1 – HFD6	LFD6 – HFD6	HFD6 – HPD6
Delta % Body weight	-0.014	-0.096	-0.049	0.168	-0.090
Delta % HbA1c	-0.063	0.036	-0.159	0.016	-0.254
Delta % Glucose	0.121	-0.150	-0.105	0.031	0.262
Delta % Insulin	0.124	-0.074	-0.074	0.038	0.315
Delta % HOMA-IR	0.147	-0.093	0.055	0.047	**0.456***
Delta % FFA	-0.057	**0.246***	0.092	0.080	-0.200
Delta % Total Cholesterol	-0.025	-0.006	**-0.229***	0.011	-0.013
Delta % LDL	-0.016	-0.004	-0.195	0.053	-0.245
Delta % HDL	-0.038	0.065	**0.381*****	-0.001	0.125
Delta % Triglycerides	-0.059	-0.023	0.002	-0.112	-0.103
Delta % IHL	n.a.	0.063	0.024	0.008	n.a.

Shown is the correlation coefficient (Spearman or Pearson according to data distribution). For the change between clinical investigation days, Scr – LFD6, LFD6 – HFD1, HFD1 – HFD6 and LFD6 – HFD6 correlations were calculated with the data of all 92 participants; for HFD6 – HPD6, the correlations were calculated with the data of the 24 participants that continued with the high-protein diet.

FFA, free fatty acid; HFD1, investigation day after high-fat diet for 1 week; HFD6, investigation day after high-fat diet for 6 weeks; HOMA-IR, homeostasis model assessment–estimated insulin resistance; HPD6, investigation day after high-protein diet for 6 weeks; IHL, intrahepatic lipid content; LFD6, investigation day after low-fat diet for 6 weeks; n.a., not available; Scr, screening.

*p < 0.05; ***p < 0.001.

Table S5: Changes in amino acids of all participants in response to high-fat diet.

	LFD6	HFD1	HFD6
Total measured AA [μM]	2223.7 ± 320.9	2229.7 ± 325.1	2239.6 ± 273.2
Ala [μM]	253.1 ± 60.2	261.3 ± 57.9	254.6 ± 56.2
Arg [μM]	71.3 ± 16.7	68.8 ± 12.9	69.3 ± 15.8
Asn [μM]	42.9 ± 7.0	41.4 ± 6.8	42.6 ± 6.1
Asp [μM]	7.3 ± 3.0	7.1 ± 2.9	7.0 ± 2.8*
Cit [μM]	23.5 ± 6.7	24.8 ± 7.0	25.0 ± 7.1
Cystine [μM]	15.3 ± 8.9	16.6 ± 9.3	15.5 ± 8.1
Gln [μM]	463.4 ± 90.9	457.4 ± 88.3	461.8 ± 80.4
Glu [μM]	121.2 ± 38.7	121.4 ± 30.2	113.9 ± 29.3
Gly [μM]	190.0 ± 55.9	195.3 ± 58.8	190.1 ± 53.3
His [μM]	76.5 ± 13.3	75.7 ± 11.2	75.6 ± 11.8
Leu [μM]	97.7 ± 19.6	98.8 ± 17.9	101.1 ± 18.6
Lys [μM]	143.7 ± 40.8	144.0 ± 34.5	146.0 ± 36.0
Met [μM]	19.1 ± 3.8	19.3 ± 3.6	19.7 ± 3.6
Orn [μM]	38.2 ± 13.4	37.3 ± 11.1	37.8 ± 14.4
Phe [μM]	45.6 ± 7.3	45.4 ± 6.2	45.9 ± 7.3
Pro [μM]	140.4 ± 51.3	157.4 ± 58.2***	146.8 ± 51.2††
Ser [μM]	93.0 ± 21.0	90.5 ± 19.8	91.4 ± 19.0
Thr [μM]	114.8 ± 32.9	109.1 ± 33.4	117.4 ± 35.5
Trp [μM]	45.6 ± 7.5	47.3 ± 8.0	47.5 ± 8.4*
Tyr [μM]	41.6 ± 10.4	43.4 ± 10.1	44.4 ± 11.1
Val [μM]	179.1 ± 60.2	183.1 ± 27.7	188.2 ± 33.2*

The values are shown as fasting mean ± SD. AA, amino acid; HFD1, investigation day after high-fat diet for 1 week; HFD6, investigation day after high-fat diet for 6 weeks; LFD6, investigation day after low-fat diet for 6 weeks.

*p < 0.05 vs. LFD6; **p < 0.01 vs. LFD6; *** p < 0.001 vs. LFD6; †p < 0.05 vs. HFD1; ††p < 0.01 vs. HFD1.

Table S6: Correlation analysis of fasting circulating amino acids with fasting glucagon.

	Glucagon			
	LFD6	HFD1	HFD6	HPD6
Total measured AA	0.092	-0.080	**0.266***	-0.171
Ala	-0.004	-0.094	**0.239***	-0.198
Arg	0.137	-0.115	0.154	-0.109
Asn	-0.044	**-0.263***	-0.119	-0.136
Asp	-0.054	-0.134	-0.028	0.162
Cit	**0.209***	-0.149	0.171	-0.212
Cystine	**0.289****	0.065	**0.296****	-0.312
Gln	-0.038	-0.089	-0.009	-0.024
Glu	0.165	0.125	**0.260***	0.343
Gly	0.116	0.022	0.164	-0.267
His	-0.087	-0.164	0.071	0.309
Leu	0.099	0.117	**0.313****	0.175
Lys	0.024	**-0.226***	0.099	0.142
Met	0.007	**-0.324****	0.127	-0.134
Orn	**0.346****	0.009	**0.256***	-0.292
Phe	0.124	0.037	**0.363****	0.256
Pro	**0.210***	0.082	**0.237***	-0.106
Ser	0.068	**-0.231***	-0.007	**-0.496***
Thr	**-0.207***	**-0.227***	-0.044	-0.402
Trp	0.024	-0.035	0.091	0.246
Tyr	**0.225***	-0.004	**0.263***	-0.099
Val	0.091	**0.228***	**0.218***	0.146

Shown is the correlation coefficient (Spearman or Pearson according to data distribution). For clinical investigation days, LFD6, HFD1 and HFD6 correlations were calculated with the data of all 92 participants; for HPD6, the correlations were calculated with the data of the 24 participants that continued with the high-protein diet. AA, amino acid; HFD1, investigation day after high-fat diet for 1 week; HFD6, investigation day after high-fat diet for 6 weeks; HPD6, investigation day after high-protein diet for 6 weeks; LFD6, investigation day after low-fat diet for 6 weeks.
*$p < 0.05$; **$p < 0.01$.

Table S7: Correlation analysis of the change (delta) in fasting glucagon with the change in fasting circulating amino acids.

	Delta Glucagon			
	LFD6 – HFD1	HFD1 – HFD6	LFD6 – HFD6	HFD6 – HPD6
Delta total measured AA	-0.204	-0.209	0.039	**-0.455***
Delta Ala	**-0.219***	-0.059	0.045	-0.106
Delta Arg	-0.129	**-0.215***	-0.070	-0.144
Delta Asn	-0.082	**-0.250***	0.005	-0.365
Delta Asp	**-0.255***	0.030	-0.034	-0.366
Delta Cit	**-0.225***	**-0.219***	-0.061	-0.370
Delta Cystine	0.173	0.003	0.104	**-0.625****
Delta Gln	-0.187	-0.096	0.087	**-0.625****
Delta Glu	0.006	0.043	-0.076	0.139
Delta Gly	-0.065	-0.078	0.013	**-0.539****
Delta His	-0.128	-0.032	0.099	0.129
Delta Leu	-0.118	-0.142	**0.229***	0.064
Delta Lys	-0.062	-0.128	-0.062	-0.214
Delta Met	**-0.245***	-0.134	**0.257***	-0.295
Delta Orn	-0.049	-0.175	-0.019	-0.328
Delta Phe	-0.100	-0.053	**0.241***	-0.035
Delta Pro	-0.066	-0.080	0.093	-0.288
Delta Ser	**-0.290****	-0.208	-0.078	-0.255
Delta Thr	-0.158	-0.206	-0.028	-0.116
Delta Trp	0.024	-0.112	0.118	-0.202
Delta Tyr	-0.097	-0.124	0.061	-0.189
Delta Val	-0.110	-0.103	0.078	-0.221

Shown is the correlation coefficient (Spearman or Pearson according to data distribution). For the change between clinical investigation days, LFD6 – HFD1, HFD1 – HFD6 and LFD6 – HFD6 correlations were calculated with the data of all 92 participants; for HFD6 – HPD6, the correlations were with the data of the 24 participants that continued with the high-protein diet.

AA, amino acid; HFD1, investigation day after high-fat diet for 1 week; HFD6, investigation day after high-fat diet for 6 weeks; HPD6, investigation day after high-protein diet for 6 weeks; LFD6, investigation day after low-fat diet for 6 weeks.

*$p < 0.05$; **$p < 0.01$.

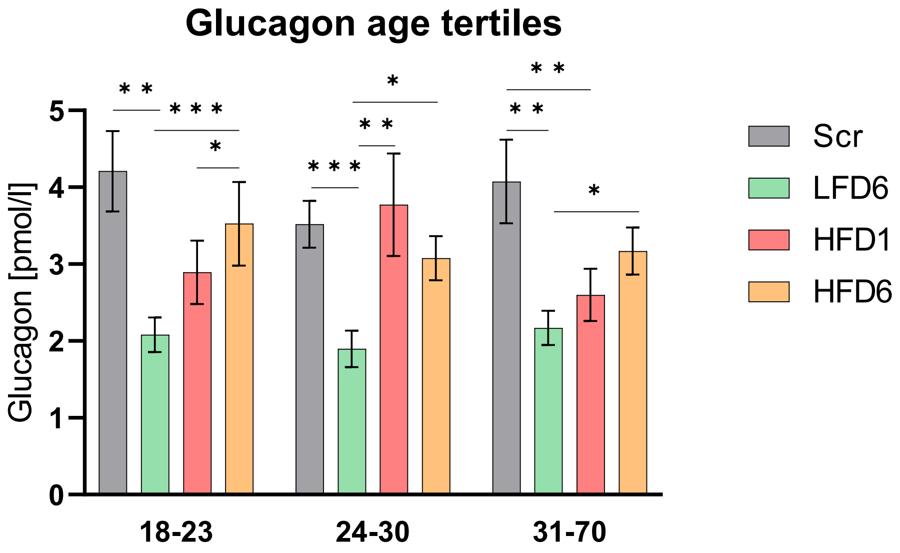

Figure S1: Glucagon changes in age subgroups. To analyze whether the age of the subjects had an impact on high-fat-diet-induced glucagon changes, the cohort was split into three age subgroups (tertiles: 1. 18-23 years, n=36 2. 24-30 years, n=26 3. 31-70 years, n=30). The values are shown as the mean ± SEM. As all groups showed a glucagon decrease with the LFD and an increase with the HFD, the induced changes in glucagon seem to occur independently of age. HFD1, investigation day after high-fat diet for 1 week; HFD6, investigation day after high-fat diet for 6 weeks; LFD6, investigation day after low-fat diet for 6 weeks; Scr, screening visit.

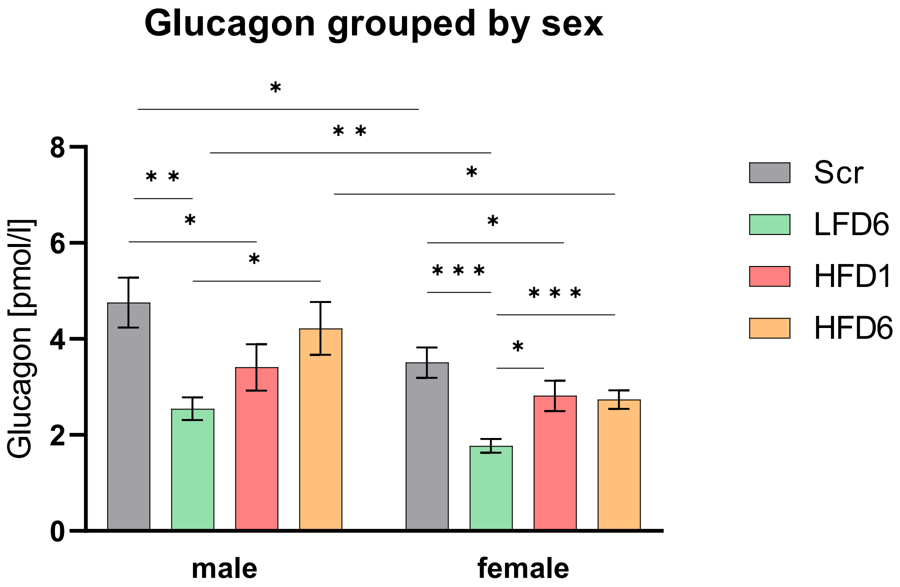

Figure S2: Glucagon changes grouped by sex. The glucagon levels were significantly higher in males (n=34) compared to females (n=58) at baseline (gray), after 6 weeks of the LFD (green) and after 6 weeks of the HFD (orange). The values are shown as the mean ± SEM. However, both sexes showed a significant glucagon decrease after 6 weeks of the LFD and a significant increase in glucagon levels in response to 6 weeks of the HFD. HFD1, investigation day after high-fat diet for 1 week; HFD6, investigation day after high-fat diet for 6 weeks; LFD6, investigation day after low-fat diet for 6 weeks; Scr, screening visit.

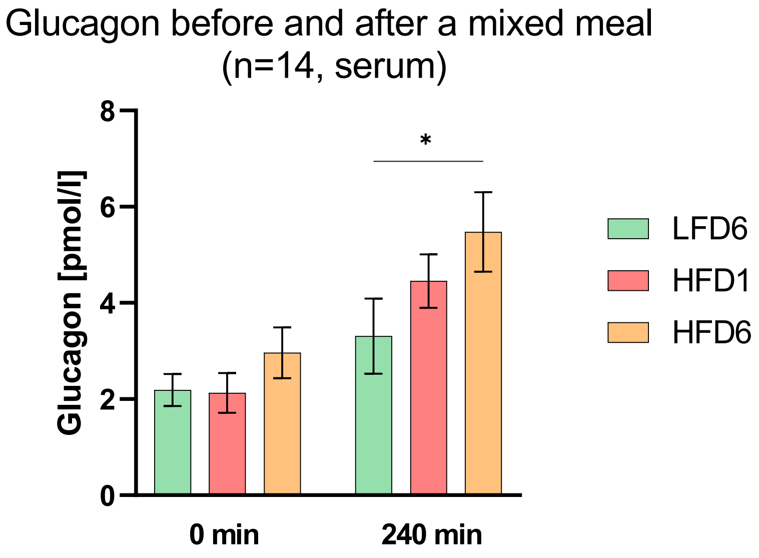

Figure S3: Glucagon levels before and after test meal. Glucagon was measured before (0 min) and after (240 min) the consumption of a test meal in a subgroup of 14 participants randomly taken from the entire study group. Repeated-measures ANOVA followed by the Bonferroni adjusted post hoc test was performed separately for each time point (0 min and 240 min). The values are shown as the mean ± SEM. In this subgroup, the glucagon levels before intake of the test meal increased slightly, but not significantly in response to the high dietary fat intake. Postprandial glucagon increased progressively and was significantly higher after 6 weeks of the HFD compared to the LFD. HFD1, investigation day after high-fat diet for 1 week; HFD6, investigation day after high-fat diet for 6 weeks; LFD6, investigation day after low-fat diet for 6 weeks.

Table S8: The characteristics of the subgroup of participants that continued with the high-protein intervention (n=24) measured on each clinical investigation day.

	Screening	LFD6	HFD1	HFD6	HPD6
Weight [kg]	72.6 ± 12.8	71.8 ± 12.1§	71.7 ± 12.2§	72.4 ± 12.5	72.5 ± 12.9
Glucose [mmol/L]	5.05 ± 0.46	5.61 ± 1.01	5.24 ± 0.71	5.49 ± 0.57	5.75 ± 0.67
Insulin [mU/L]	5.38 ± 4.00	5.25 ± 4.01	6.19 ± 5.05	5.88 ± 4.41	4.07 ± 2.92†
HOMA-IR	1.07 ± 0.76	1.33 ± 1.08	1.42 ± 1.26	1.45 ± 1.13 §§	1.03 ± 0.72#
Glucagon [pmol/L]	3.21 ± 2.75	2.10 ± 1.12	2.99 ± 2.09	3.43 ± 2.48*	4.55 ± 1.49 §*** †††
FFA [mmol/L]	0.47 ± 0.22	0.58 ± 0.25	0.54 ± 0.19	0.46 ± 0.16	0.60 ± 0.14§
TC [mmol/L]	4.69 ± 0.98	4.38 ± 0.92§	4.41 ± 1.05	4.82 ± 1.05**	4.38 ± 0.81#
LDL [mmol/L]	2.89 ± 0.83	2.74 ± 0.76	2.71 ± 0.86	2.97 ± 0.91	2.73 ± 0.66
HDL [mmol/L]	1.32 ± 0.34	1.18 ± 0.29 §§§	1.21 ± 0.36	1.36 ± 0.42 ***†	1.22 ± 0.32§##
TG [mmol/L]	0.94 ± 0.38	1.01 ± 0.45	0.87 ± 0.33	0.92 ± 0.42	0.94 ± 0.68

The values are shown as fasting mean ± SD. FFA, free fatty acid; HFD1, investigation day after high-fat diet for 1 week; HFD6, investigation day after high-fat diet for 6 weeks; HOMA-IR, homeostasis model assessment–estimated insulin resistance; HPD6, investigation day after high-protein diet for 6 weeks; LFD6, investigation day after low-fat diet for 6 weeks; TC, total cholesterol; TG, triglyceride

§p < 0.05 vs. screening; §§p < 0.01 vs. screening; §§§p < 0.001 vs. screening; *p < 0.05 vs. LFD6; **p < 0.01 vs. LFD6; *** p < 0.001 vs. LFD6; †p < 0.05 vs. HFD1; ††p < 0.01 vs. HFD1; ††† p < 0.001 vs. HFD1; # p < 0.05 vs. HFD6; ## p < 0.01 vs. HFD6; ###p < 0.001 vs. HFD6.

Table S9: Changes in fasting amino acids in the subgroup of participants that continued with the high-protein intervention (n=24).

	LFD6	HFD1	HFD6	HPD6
Total measured AA [µM]	2360.8 ± 205.3	2380.3 ± 239.7	2335.5 ± 258.6	2353.2 ± 239.1
Ala [µM]	256.2 ± 61.6	277.6 ± 56.6	260.8 ± 67.2	232.4 ± 54.4*†††##
Arg [µM]	78.1 ± 16.0	70.7 ± 14.3	72.7 ± 12.3	71.8 ± 12.5
Asn [µM]	42.6 ± 6.9	39.2 ± 6.6	39.9 ± 5.5	41.2 ± 6.1
Asp [µM]	8.6 ± 3.1	8.0 ± 3.3	7.8 ± 3.3	9.7 ± 3.1
Cit [µM]	24.1 ± 6.2	24.6 ± 6.7	24.2 ± 7.4	27.6 ± 9.1
Cystine [µM]	16.7 ± 7.4	18.6 ± 8.8	15.4 ± 7.0	18.0 ± 7.3
Gln [µM]	509.6 ± 70.7	509.6 ± 83.8	484.8 ± 77.8	449.7 ± 65.6**††
Glu [µM]	123.8 ± 36.1	124.9 ± 32.4	112.9 ± 24.1†	125.1 ± 26.7#
Gly [µM]	205.1 ± 45.8	218.5 ± 59.1	205.1 ± 42.5	178.4 ± 40.2*††#
His [µM]	80.3 ± 10.2	75.2 ± 10.4	77.1 ± 12.7	79.0 ± 11.1
Leu [µM]	102.5 ± 14.5	101.4 ± 16.9	103.7 ± 18.2	114.5 ± 21.1*†
Lys [µM]	160.8 ± 30.3	161.6 ± 34.9	164.2 ± 32.7	182.8 ± 38.6*†
Met [µM]	20.9 ± 3.5	20.6 ± 3.7	20.2 ± 4.0	23.4 ± 4.0#
Orn [µM]	42.4 ± 11.8	39.7 ± 9.7	43.5 ± 20.4	41.6 ± 11.9
Phe [µM]	46.4 ± 4.6	44.8 ± 6.0	44.7 ± 6.2	51.1 ± 4.5**††##
Pro [µM]	156.2 ± 61.4	177.0 ± 69.5*	167.8 ± 65.2	156.8 ± 65.7†
Ser [µM]	94.3 ± 17.7	90.6 ± 21.1	92.9 ± 20.8	97.2 ± 14.6
Thr [µM]	108.2 ± 33.4	99.6 ± 33.9	102.5 ± 25.7	114.3 ± 26.9†
Trp [µM]	47.3 ± 8.3	47.1 ± 7.8	47.9 ± 9.7	55.5 ± 8.6**††#
Tyr [µM]	46.5 ± 8.4	46.8 ± 8.9	50.3 ± 10.6	56.3 ± 10.3
Val [µM]	190.3 ± 30.0	184.1 ± 27.9	197.2 ± 36.4	226.6 ± 32.6**†††##

The values are shown as fasting mean ± SD. AA, amino acid; HFD1, investigation day after high-fat diet for 1 week; HFD6, investigation day after high-fat diet for 6 weeks; HPD6, investigation day after high-protein diet for 6 weeks; LFD6, investigation day after low-fat diet for 6 weeks; *p < 0.05 vs. LFD6; **p < 0.01 vs. LFD6; *** p < 0.001 vs. LFD6; †p < 0.05 vs. HFD1; ††p < 0.01 vs. HFD1; ††† p < 0.001 vs. HFD1; # p < 0.05 vs. HFD6; ## p < 0.01 vs. HFD6; ###p < 0.001 vs. HFD6.

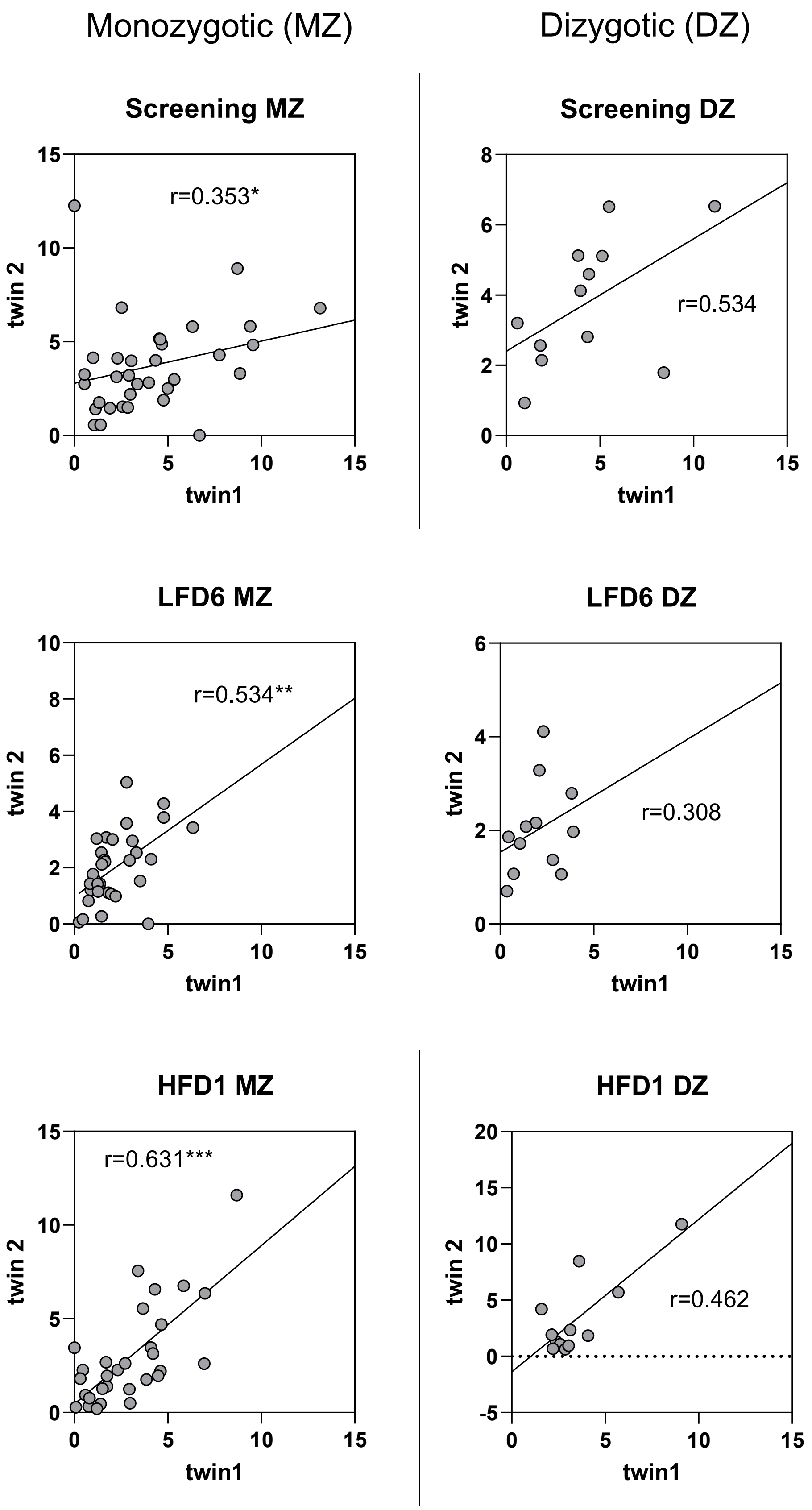

A:0.000
C:0.353
E:0.647

A:0.452
C:0.082
E.0.466

A:0.338
C:0.293
E:0.369

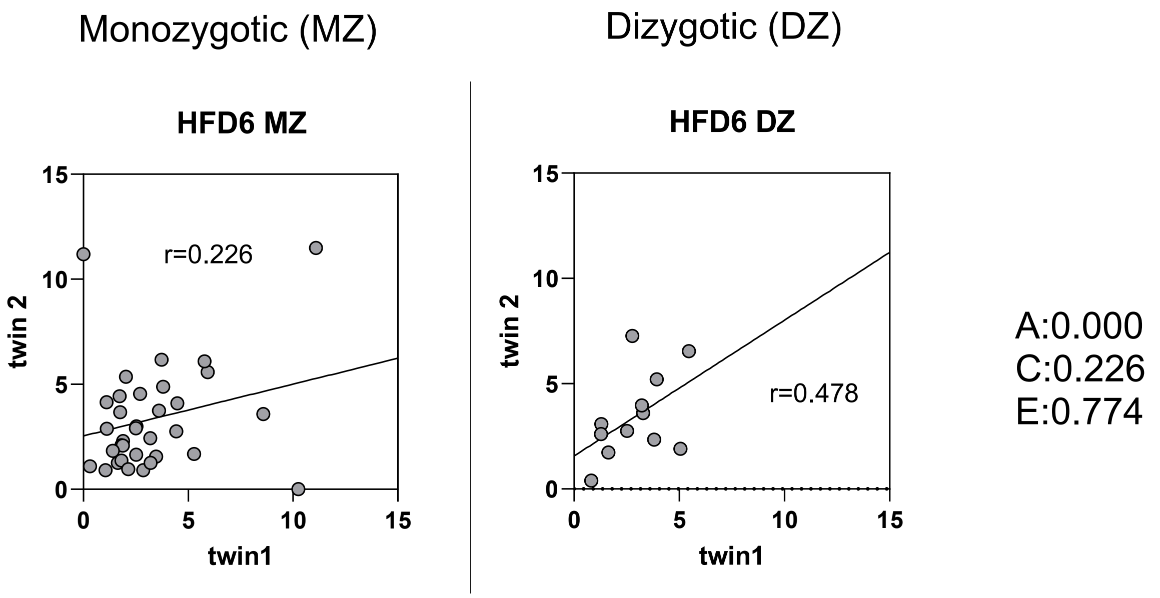

Figure S4: Heritability of glucagon. Heritability was estimated by applying the “ACE structural equation model” for each clinical investigation day (CID). The x and y axis show plasma glucagon in pmol/L. A, additive genetic influence; C, common environmental influence; E, individual environmental influence; HFD1, investigation day after high-fat diet for 1 week; HFD6, investigation day after high-fat diet for 6 weeks; LFD6, investigation day after low-fat diet for 6 weeks.
